# High risk human papilloma viruses (HPVs) are present in benign prostate tissues before development of HPV associated prostate cancer

**DOI:** 10.1186/s13027-017-0157-2

**Published:** 2017-08-11

**Authors:** Wendy K. Glenn, Christopher C. Ngan, Timothy G. Amos, Richard J. Edwards, Joshua Swift, Louise Lutze-Mann, Fei Shang, Noel J. Whitaker, James S. Lawson

**Affiliations:** School of Biotechnology and Biomolecular Science, University of New South Wales, Sydney, NSW Australia

**Keywords:** Prostate cancer, Benign prostate, Biopsy, Human papilloma virus, HPV E7, Cytokeratin, Prostate specific antigen, Koilocytes, PCR, RNA-Seq, Immunohistochemistry

## Abstract

**Background:**

Although high risk HPVs are associated with an increased risk of prostate cancer it is not known if they have a causal role. The purpose of this study is to investigate the potential role of human papilloma viruses (HPVs) in prostate cancer. The aims are (i) to investigate the presence and confirm the identity of high risk HPVs in benign prostate tissues prior to the development of HPV positive prostate cancer in the same patients, and (ii) to determine if HPVs are biologically active.

**Methods:**

We used polymerase chain reaction (PCR) to identify HPVs in specimens from 52 Australian men with benign prostate biopsies who 1 to 10 years later developed prostate cancer. Immunohistochemistry (IHC) was used to assess the expression of HPV E7 oncoproteins, cytokeratin and prostate specific antigen (PSA).

We used RNASeq data from The Cancer Genome Atlas (TCGA) to identify possible HPV RNA sequences in prostate cancer.

**Results:**

HPV screening using standard PCR was conducted on 28 of the 52 sets of benign and later prostate cancers. HPV L1 genes were identified in 13 (46%) benign and 8 (29%) of 28 later prostate cancers in the same patients. HPV E7 genes were identified in 23 (82%) benign and 19 (68%) of 28 subsequent prostate cancers in the same patients. The same HPV types were present in both the benign and subsequent prostate cancers in 9 sets of specimens. HPV type 16 was identified in 15% of benign and 3% of prostate cancers. HPV type 18 was identified in 26% of benign and 16% of prostate cancers. Small numbers of HPV types 45, 47, 76 and 115 were also identified.

High confidence RNA-Seq evidence for high risk HPV types 16 and 18 was identified in 12 (2%) of the 502 TCGA prostate cancer transcriptomes.

High risk HPV E7 oncoprotein was positively expressed in 23 (82%) of 28 benign prostate specimens but only in 8 (29%) of 28 of the later prostate cancer specimens. This difference is statistically significant (*p* = 0.001). Prostate specific antigen (PSA) was more highly expressed in 26 (50%) of 52 prostate cancer specimens as compared to prior benign prostate specimens in the same patients.

**Conclusions:**

High risk HPVs are present in benign prostate tissues prior to the development of HPV positive prostate cancer. There is a significantly higher expression of HPV E7 oncoproteins in benign prostate tissues as compared to late prostate cancer that subsequently developed in the same patients. This observation suggests that HPV oncogenic activity is an early phenomenon in a majority of prostate oncogenesis. TCGA RNA-Seq data suggests that HPV is biologically active in some prostate tumour samples.

**Electronic supplementary material:**

The online version of this article (doi:10.1186/s13027-017-0157-2) contains supplementary material, which is available to authorized users.

## Background

Human papilloma viruses (HPVs) with high risk for cancer have been identified in prostate cancers in men located in North and South America, Europe and the Asia/Pacific region including Australia and New Zealand [1]. The most frequently identified high risk HPVs in prostate cancers are types 16, 18, 31, 33 and 58. Meta-analysis of 32 PCR-based studies concluded that high risk HPVs are associated with an increased risk of prostate cancer with an odds ratio of 1.8 [1, 2]. Although there is limited data, up to 22.3% of men with an initial negative prostate biopsy develop prostate cancer within 11 years [3–5]. One of the criteria for evidence of causation of a disease by a pathogenic agent is the presence of that agent in normal or benign tissues prior to the development of the disease [6]. However, while HPVs have been identified in benign prostate tissues, no data is available from the same patients to confirm or refute causality based on this criterion.

Many authors of past studies express the view that HPVs probably do not have a causal role in prostate cancer because (i) there is a similar prevalence in several studies of high risk HPVs in benign and malignant prostate tissues, (ii) the lack of an association of HPV antibodies and prostate cancer in most serological studies, (iii) the failure of next generation sequencing to identify HPVs in prostate cancers [7–11].

Because the validity of these adverse views is not clear we have undertaken this current study. The aims are (i) to investigate the presence of high risk HPVs in benign prostate tissues prior to the development of HPV positive prostate cancer in the same patients and (ii) to determine if high risk HPVs are biologically active and not mere harmless “passenger” viruses in prostate tissues.

## Methods

### Patients and samples

Patients who had an initial benign prostate biopsy and subsequently developed prostate cancer were identified from the files of Douglass Hanly Moir Pathology Sydney, Australia. The first step in the identification of these patients was made by reviewing pathology reports which indicated the presence of prostate cancer. The next step was to identify those patients who previously had benign prostate biopsies. These patient tissue samples were considered eligible for this study without additional selection criteria. Neither ethnic nor racial characteristics are recorded for Australian patients. The samples were archival formalin fixed and paraffin mounted specimens. Fifty-two sets of benign and prostate cancer specimens from the same patients were identified and collected.

### Identification of HPV gene sequences by polymerase chain reaction (PCR)

Standard PCR and semi-nested PCR were used for the detection of HPVs. L1 PCR products were used to identify HPV types. Formalin fixed paraffin embedded tissues (FFPE) were deparaffinised with Qiagen deparaffinising solution, then genomic DNA was isolated using Qiagen DNA FFPE tissue kit. All procedures were conducted in accord with the manufacturer’s instructions. For standard PCR, the primers used are shown in Table 1. Thermal cycles for all PCR reactions were: 94 °C for 15 min; 94 °C for 30 s, 55 °C for 30 s, 72 °C for 45 s for 30 cycles. HotStarTaq Master Mix Kit (QIAGEN) was used for the PCR reaction master mix. Prior to HPV screening, genomic DNA samples were amplified with ß-actin using standard PCR. Samples that were ß-actin positive were selected for HPV screening. Semi-nested PCR was used to detect and amplify the HPV L1 gene; the primers used in the first round PCR were MY11 and GP6+. After the first round PCR, one microliter of the amplified product was added to a new tube containing the master mix with Gp5+ to Gp6+ primers for the second round PCR. These primers were degenerate for HPV16 and 18, but were also capable of bringing up types 3, 11, 12, 45, 47, 58, 73, 75, 76 and 115.

**Table 1:** PCR primers used for standard and semi-nested PCR

*Primers*	*Primer sequence (5’➔ 3′)*	*Target*
ß-Globin forward	GAAGAGCCAAGGACAGGTAC	ß-globin
ß-Globin reverse	CAACTTCATCCACGTTCACC	
MY11^b^	GCACAGGGYCAYAAYAATGG	HPV L1
Gp6 + ^c^	AATCATATTCCTCMMCATGTC	
Gp5+ ^b^	TATTTGTTACTGTKGTWGATAC	
HPV18 E7 forward	GACGAGCCGAACCACAAC	HPV18 E7
HPV18 E7 reverse	GGATGCACACCACGGACA	
HPV16 E7 forward	AGCTCA GAGGAGGAGGATGA	HPV16 E7
HPV16 E7 reverse	GGTTTCTGAGAACAGATGGG	

^a^Degenerate bases: M = A + C, W = A + T, Y = C + T, R = A + G

^b^Forward primer

^c^Reverse primer

Genomic DNA extracts were also tested for the presence of an HPV-E7 gene using sub-type specific (HPV-16 and -18 E7) primers. Production of the HPV E7 gene consisted of two 30 cycles of PCR reactions. After the first round of PCR, one microliter was subjected to a second-round PCR reaction, using the same primers, for an additional 30 cycles.

Stringent negative controls were used in parallel with all PCR analyses. These negative controls were no DNA (water) and a reagent blank from the extraction procedure, plus sequencing of the products of these controls in case the bands could not be seen on a gel. Positive controls for HPV were an HPV 18 positive cell line (HeLa), and HPV 16 positive cell line (SiHa).

### Sequencing the PCR products and identification of HPV types

Standard PCR was used to screen all samples with HPV E7 primers. Amplified PCR products were then visualized by gel electrophoresis. Samples positive for E7 were sequenced to validate the authenticity of the PCR products. GP5+ and GP6+ primers that target the subsection of the HPV L1 gene were sequenced to determine the HPV type. The HPV genotypes were identified by BLAST via the US National Center for Biotechnology Information (NCBI).

### The cancer genome atlas (TCGA) RNA-Seq bioinformatics analysis

Viral genomes for the initial screen were downloaded from the NCBI Genome database (December 2014: 4742 distinct virus names including 50 HPV strains (Additional file 1: Table S1A) [12]. A custom Python script was used to fix some strand issues in the accompanying gff file using the equivalent NCBI Reference Sequence (Ref-Seq) (Release 67, December 2014) [13] virus gff file. Paired-end (PE) RNA-Seq data for 502 prostate cancer transcriptomes and 52 normal prostate controls were downloaded from TCGA in BAM format. BAM files were converted into interleaved fastq format using bedtools [14] v2.19.0 and split into forward and reverse reads using a custom Python script.

In the initial screen reads were aligned to all NCBI viral genomes using tophat [15] v2.0.13, the options *--no-novel-juncs --read-mismatches 4 --read-edit-dist 4* and the corresponding gff file. Reference files were indexed using Bowtie2 [16] v2.2.4. SAMtools [17] v1.1 and Picard tools (http://sourceforge.net/projects/picard/) v1.109 were used to extract all reads that aligned to any virus and their pairs. PythonViral reads (and their PE mates) were mapped to the human genome (Ensemble GRCh37) with tophat, the *--no-novel-juncs* option and the corresponding gff file. The same reads were also mapped to the UniVec database (*N* = 4626, November 2014, http:// ncbi.nlm.nih.gov/tools/vecscreen/univec) with tophat using *--min-anchor-length 100* to prevent intron junctions. Reference files were indexed using Bowtie 2 [2, 3, 16] v2.1.0. BAM to SAM format conversion was done using SAMtools. Viral reads from PE pairs for which neither read hit human or Univec were designated “filtered reads”.

The complexity of reads was assessed by its compression ratio using the Python package zlib. High complexity was chosen as not compressible below <50% of its length (excluding eight header characters). Reads were assessed for quality using the mean of their Phred-like quality scores. Thus, all hits to virus genomes could be assessed for reliability using matches to the human genome or vector database, by their coverage, quality, by the complexity of the reads, whether they mapped to multiple viruses and whether both reads in a PE pair mapped to the same virus. Custom Python scripts were used to create consensus sequences from the SAM files (Additional file 1) and read counts per gene from the SAM and gff files.

Viruses that had reads aligned to them in each sample were filtered to create a set enriched with higher confidence detections. Filtered reads were labelled “high quality” if they had a minimum average quality score of Q30, a minimum complexity (compressibility) of 50% and a maximum edit distance of two. To be considered high confidence: (i) at least 40% of “high quality” filtered reads constitute “high quality read pairs”, where both PE reads are high quality and map to the same virus; (ii) at least 192 bp must be covered by high quality read pairs. (Since the reads were 48-50 bp long, requiring 192 bp of coverage meant requiring at least four reads with no overlaps between them, for the shorter read lengths.) Enrichment of HPV in the higher confidence set was calculated by comparing the fraction of all possible HPV virus-sample combinations in which reads were found that aligned to an HPV to the equivalent fraction for non-HPV viruses in the unfiltered and higher confidence sets.

### Immunohistochemistry (IHC)

Antibodies, that are specific for HPV E7 proteins, have recently been developed [18, 19]. These antibodies (Cervimax) were used in this study. The specificity of these antibodies has been demonstrated experimentally and by epidemiological studies [18–20]. The HPV E7 antibody reacts with a wide range of HPV types including high risk for cancer HPV 16 and 18. Good outcomes for HPV E7 were achieved with clear staining of both cytoplasm and nuclei of prostate cancer cells with 1 to 100 dilution of the antibody without antigen retrieval. Standard manual IHC methods were used for HPV E7, with the omission of the antigen retrieval step. The antibodies were HPV E7 monoclonal “Cervimax” - Valdospan GmbH. Austria. Positive controls for the E7 antibody were cervical tissues that were positive by PCR and sequencing. Freshly cut slides needed less antibody (1/100 for 30 mins) than recommended by the manufacturer (Valdospan). Slides that were up to 5 years old needed 1/100 dilution of antibody for 2 h. HPV E7 staining was assessed on a scale of 0 to 1, with 0 indicating a negative result and 1 a positive result.

Antibodies, specific for prostate specific antigen (PSA) and cytokeratin, were used to assess the expression of PSA and cytokeratin in the archival formalin fixed benign prostate and prostate cancer specimens. Automated IHC methods used a Bond-RX automatic staining system (Bond polymer refine, Cat #DS9800) Leica biosystems. The intensity of PSA expression was assessed on a sliding scale of 0 to 3. Cytokeratin was assessed on a scale of 0 to 1, where 0 indicates loss of basal staining due to cellular degradation and 1 indicates intact (normal) or semi-intact (hyperplasia) cellular structure.

#### Statistics

A McNeumar’s test for categorical data was used to compare the proportions of benign and prostate cancer specimens according to whether HPV genes only or HPV protein only or both HPV genes and protein were present in the same specimen. The statistical test was carried out using IBM SPSS Statistics (ver.19). The tests were all two-sided, and statistical significance was defined as *p* ≤ 0.05.

## Results

### Identification of HPVs by PCR

HPV screening using standard PCR was conducted on 28 of the 52 sets of benign and later prostate cancers. High risk HPV L1 genes were identified in 13 (46%) benign and 8 (29%) of later prostate cancers in the same 28 patients (Table 2). HPV E7 genes were identified in 23 (82%) benign and 19 (68%) of later prostate cancers in the same 28 patients (Table 2). The same HPV types were present in both the benign and subsequent prostate cancers in 9 (32%) sets of 28 patient specimens. Because some formalin fixed paraffin embedded (FFPE) tissues were insufficient or could not amplify beta globin, outcomes based on PCR, were not obtained for either the prior benign or subsequent prostate cancers in specimens from 24 patients. However, there was adequate material to conduct immunohistochemistry on all 52 sets of specimens.

**Table 2:** Presence of high risk HPV L1 and E7 viral genes and HPV E7 protein in benign prostate and prostate cancer (same sets of patients)

	Benign prostate with subsequent prostate cancer	Prostate cancer	Difference in proportions between benign prostate and prostate cancer
HPV L1 gene	13/28 (46%)	8/28 (29%)	× ^2^ = 1.231 df = 1 *p* = 0.267 (ns)
HPV E7 gene	23/28 (82%)	19/28 (70%)	× ^2^ = 0.750 df = 1 *p* = 0.387 (ns)
HPV E7 protein	23/28 (82%)	8/28 (29%)	× ^2^ = 11.529 df = 1 p = <0.001 (sig)

There were no statistically significant differences in the prevalence of HPV L1 and HPV E7 genes (as assessed by PCR) between prior benign prostate and later prostate cancer in the same patients. The differences in HPV E7 oncoprotein expression (as assessed by immunohistochemistry) were highly significant with much higher expression in the benign as compared to the later prostate cancer in the same patient

A two-tailed McNemar test was used to assess the statistical significances

× ^2^ = chi-square, d.f = degree of freedom. Sig = significant, ns = not significant

There were both similarities and differences in outcomes based on either PCR L1 or PCR E7 primers. The details are shown in Additional file 1: Table S1. The identification of HPV types was based on sequencing the PCR L1 products. HPV type 16 was identified in 5 (15%) of 34 benign and 1 (3%) of 32 prostate cancers. HPV type 18 was identified in 9 (26%) of 34 benign and 5 (16%) of 32 prostate cancers. HPV type 45 (3%) was identified in one prostate cancer specimen. HPV type 47 was identified in 3 (9%) benign and 2 (6%) prostate cancers. HPV type76 (6%) was identified in 2 benign prostate specimens. HPV type 115 (3%) was identified in one benign prostate specimen.

The identification of specific HPV types 16 and 18 based on the PCR E7 products was (i) benign prostate specimens HPV type 16: 20 (63%) of 32 specimens, HPV type 18: 16 (50%) of 32 specimens, (ii) prostate cancer specimens HPV type 16: 11 (34%) of 32 specimens, HPV type 18: 13 (41%) of 32 specimens.

**Fig. 1:**
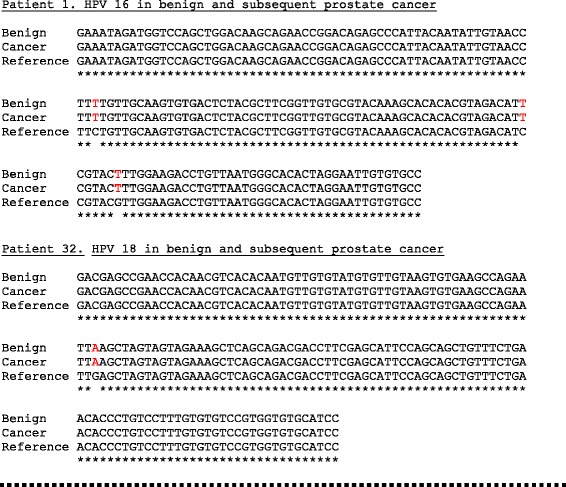
Identical HPV type 16 and 18 E7 gene sequences in benign and subsequent prostate cancer in two-selected patients. There are sequence variations which are identical in both the benign and subsequent prostate cancers which were diagnosed 2 (patient 1) and 6 (patient 32) years later. The reference sequences were HPV 16 (AF4020678) and HPV 18 (AY262282). The primer sequences have been omitted. The implication of these observations is that the same specific HPV virus was identified in both the benign and later prostate cancer in the same patient

The sequencing results from a set of selected prostate tissue samples that were positive for HPV E7 were compared with reference sequences are shown in Fig. 1. There were a small number of sequence variations in both the benign and subsequent prostate cancers which were diagnosed 2 years (patient 1) and 6 years (patient 32) later (Fig. 1). The presence of sequence variations which differ from the reference sequences indicate that contamination during PCR preparation is unlikely.

### Identification of HPVs by immunohistochemistry

**Fig. 2:**
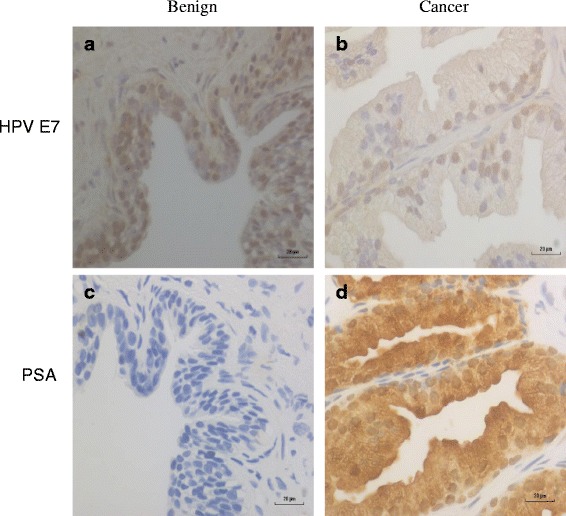
Benign and subsequent prostate cancer in the same patient screened with HPV E7 and PSA. There is higher HPV protein expression in benign prostate tissues (**a**) as compared to subsequent prostate cancer (**b**) in the same patient. There is no PSA expression in benign prostate tissues (**c**) as compared to high expression in subsequent prostate cancer in the same patient (**d**)

Positive HPV E7 oncoprotein expression was present in 40 (76.9%) of 52 benign specimens and in 13 (25%) of 52 prostate cancer specimens. In the 28 sets of benign and later prostate cancer specimens (same patients) in which HPV gene sequences were identified, HPV E7 oncoprotein was expressed in 23 (82%) of benign and 8 (29%) of later prostate cancers (Table 2). This striking difference in HPV E7 oncoprotein expression between HPV positive benign and HPV positive prostate cancer is statistically significant (*p* = 0.001). HPV E7 expression in benign and later prostate cancer in the same patient is shown in Fig. 2.

### Identification of HPVs from RNA-Seq data

High risk HPV types 16 and 18 were putatively identified with high confidence in RNA-Seq data for 12 out of 502 prostate cancer samples in TCGA. A further seven tumour samples and one (of 52 normal) prostate control samples had lower confidence evidence for HPV of which 4/7 tumour hits were also HPV 16/18. These data are shown in Table 3 (and Additional file 1: Table S1B) where 10/12 (83%) high confidence and 14/17 (82%) total samples with HPV (16 or 18) hits had evidence of HPV E7 gene expression.

**Table 3:** HPV DNA identification in the TCGA series of prostate cancers

Sample ID	HPV type	E6	E7	E1	E2	E4	E5	L2	L1	Confidence
df64c493-d290-41c7-abd3-ab65cda57b02	16	8	12	5	37	24	16	2	0	High
5faf8ec8-f94c-4b0c-9d91-70942f15f3c8	16	3	5	8	50	28	14	0	0	High
b13d4e3d-898e-4339-a506-110b8e803b6e	16	6	9	1	18	10	5	0	0	High
2d8b33e3-002b-4592-9bc3-b3eab242c893	16	0	3	4	14	10	8	0	0	High
627b9ff8–2557-4470-824a-b76a8bcd1e9f	16	8	12	1	0	0	0	0	6	High
3ea3bcfc-2030-4d0b-b9b2-840a9ad1acaf	16	1	3	2	7	5	6	0	0	High
bccb7f9d-8dde-45b7-a50d-6c7233239ceb	18	3	4	12	0	0	0	0	0	High
7413d045-cedb-4ec6-95ea-6f7b676f800d	16	0	0	0	11	6	7	0	0	High
16,410,934-a686-4dbd-9086-bc060135f0b4	16	0	1	0	11	4	4	0	2	High
6c728891–8924-4a51-b3ed-f4b83d6c8de4	16	1	2	2	7	3	1	0	0	High
4da83354–4512-4dca-af59-dc8217363511	16	0	0	1	3	1	2	2	0	High
09bb6311-41ad-46a1–8195-6d1fdacd7cb0 ^a^	16	0	4	0	0	0	0	0	0	High
ebb062c0-ea5b-48f6-aeee-2674b6dabdec^a,b^	16	2	1	2	0	0	0	0	0	Med
dcceaf54-0bce-4650-9980-9843abce0c41^a^	16	2	0	0	0	0	0	0	0	Med
f9160e70-27f0-4c8c-a667-b24195ad659c	16	0	2	0	0	0	0	0	0	Med
563e009c-c93d-46 cm^3^-867d-f5c89d9336d9	48	0	0	2	0	0		0	0	Med
1b59308e-48 cm^3^-4bd9-b990-c82154487522	4	0	0	2	0	0		0	0	Med
4cd7f57c-fd19-4def-a5fb-dca3dbc10320	16	1	3	2	5	4	0	0	0	Low
53ebd660-7f5d-433a-acb7-4153f36484d0	18	1	1	0	0	0	0	0	0	Low
aa0e444b-c913-4a70-a76d-be70ccf80691	96	0	0	0	0	0		1	0	Low

HPV types and genes (E6, E7, E1, E2, E4, E5, L2, L1) identified by Next Generation Sequencing. 0 indicates no reads. Of the 17 HPV 16/18 positive prostate cancer samples 14 (82%) had reads for the E7 gene

^a^marks TGCA specimens with HPV reads from Tang et al. 2013

^b^marks normal tissue. Confidence is marked at high (both quality filters), Med (one quality filter), Low (no quality filters)

In total, there were 107,253 virus hits from across the 554 prostate samples (Additional file 1: Table S1C), including 87 to various HPV strains. Most of these are likely to be false positives arising from similarity to human genome sequences and/or contamination by common vector in molecular biology. Reads were therefore filtered to PE read pairs for which neither read aligned to the human or Univec references (see Methods).

Filtered reads that aligned to HPV 16 were found in 15 out of 502 prostate cancer samples and 1 out of 52 prostate control samples. Additionally, reads were found that aligned to HPV 18 in two cancer samples, HPV 4 in one sample, HPV 48 in one sample and HPV 96 in one sample (Additional file 1: Table S1B). 39,483 hits were also found for other viruses, which corresponded to approximately 1.5% of all possible viral-sample pairs. Almost all of these viral hits were probably false positives based on poor read mapping, so additional filters of read confidence and coverage were added. These reduced the total number of viral hits to 111 (0.004% of all possible hits) (Additional file 1: Table S1D), of which 12 were high risk HPV viruses (11 HPV 16 and one HPV 18) (Additional file 1: Table S1B). This represents an approximate 11-fold enrichment of HPV over that predicted by other viruses (assuming they are false positives) (Additional file 1: Table S1E) or 287-fold enrichment if just HPV16/18 are considered. Also of note, this higher confidence set contained three samples with reads aligning to human herpes virus 4 (Epstein Barr virus).

### Prostate specific antigen (PSA) expression

Prostate specific antigen (PSA) was expressed in 41/52 (79%) of benign prostate and 46/52 (88%) of prostate cancer specimens (Additional file 2: Table S2).

When comparing benign to cancer PSA levels for the same patient, 26/52 (50%) were more highly expressed, 12/52 (23%) were equally expressed and 14/52 (27%) were more lowly expressed (Additional file 2: Table S2). PSA expression in benign and later prostate cancer in the same patient is shown in Fig. 2. The implication is that PSA expression is frequently, but not consistently, associated with prostate cancer.

### Cytokeratin protein expression

**Fig. 3:**
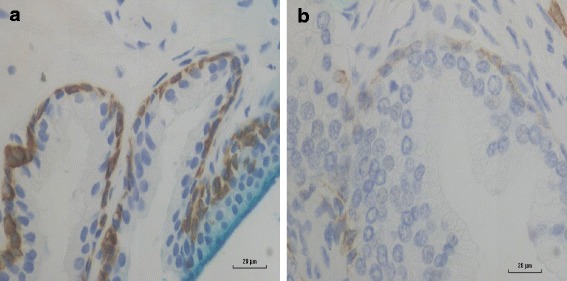
Cytokeratin staining in benign and subsequent prostate cancer which developed in the same patient 2 years later. Panel **a** Benign prostate basal cells showing strong cytokeratin immunoreactivity (brown staining). Panel **b** Prostate cancer cells in the same patient showing weak and absent cytokeratin immunoreactivity. This pattern of changes in cytokeratin staining and absence of basal cells as prostate cancer develops is useful in the diagnosis of prostate cancer

Cytokeratin protein expression can be used as a diagnostic marker of prostate in 43 (83%) of 52 benign prostate specimens and only 20 (38%) of prostate cancer specimens (*p* = 0.001). This data is shown in Additional file 2: Table S2. The expression of cytokeratin in benign and later prostate cancer in the same patient is shown in Fig. 3. These observations confirm the value of cytokeratin as a helpful marker of prostate hyperplasia and cancer with low or absent expression an indication of hyperplasia or malignancy.

## Discussion

The results of this retrospective cohort study indicate (i) that high risk HPVs, predominantly HPV 16 and 18, are commonly present in benign prostate tissues 1 to 10 years prior to the development of HPV positive prostate cancer and (ii) there is a much higher prevalence of HPV E7 oncoprotein expression in benign prostate tissues as compared to subsequent prostate cancer specimens in the same patient. While high risk HPVs have previously been identified in benign prostate tissues [3–5] there appear to be no previous reports of the identification of HPVs in benign prostate tissues prior to the development of HPV positive prostate cancer *in the same patients.* This observation is one of the key criteria for evidence of causation by a pathogenic agent [6]. In addition, these observations suggest that high risk HPVs may have an early oncogenic influence in the development of many prostate cancers. Further, these observations may explain why there is no increase in prevalence of HPV positive prostate cancer as would be expected in immunocompromised patients (such as patients with HIV or organ transplantation).

The use of a range of techniques namely PCR with two different primers, RNA-Seq data, and immunohistochemistry, have given broadly similar outcomes. However, the identification of HPVs from RNA-Seq data is much lower than their identification by PCR techniques. One possible explanation is that PCR amplification techniques are more sensitive for interrogating a relatively small number of transcripts than the current depth of sequencing employed by TCGA studies. The use of RNA-Seq data could also have excluded some HPV infections that are now transcriptionally silent [21].

Whilst ongoing viral infections (e.g. HPV in cervical cancer) can be easily detected in the cancer transcriptome, in other situations viruses may contribute to cancer causation without sustained high expression levels. We therefore analysed TCGA prostate cancer transcriptomes for evidence of both high and low expression levels of HPV. Previous studies [10] have filtered out all reads that might not be viral and then set thresholds for real infections by comparing to positive controls of ongoing infections, thereby performing high stringency searches for ongoing infections [10]. Since we were looking for evidence of viral oncogenesis regardless of ongoing expression levels we took a different approach of minimising false negatives during our initial search for candidate viral expression, and then applying a set of filters and quality controls to assess the strength of a given candidate HPV-containing sample. By screening cancer transcriptomes against several thousand viruses in the NCBI database, including non-human viruses, we were able to compare the strength of evidence against known false positives and random hits.

The finding of a variety of HPV types, suggests these HPV sequences were derived from patient samples and not contaminants. HPV E6 and E7 gene expression potentially identified by RNA-Seq add validity to the outcomes based on immunohistochemistry.

However, Tang et al. found small numbers of HPV 16 reads in three samples from TCGA (two tumours and one normal tissue). The most likely reason for the smaller number of HPV 16 identifications by Tang et al. as compared to this current study is the smaller number of samples (140 primary solid tumour and 39 solid tissue normal) [10]. In contrast, we examined 554 (502 primary solid tumours, 52 solid tissue normal and one metastatic). Of the three samples that Tang et al. [10] found HPV 16 reads in, we found the same number of reads in two samples and an additional read (that didn’t survive filtering) in a third. These samples were among those where we found lower numbers of HPV 16 reads and only one made it into our “high confidence” set. This implies that if more samples had been available at the time, Tang et al. would have found HPV 16 reads in more samples and in higher numbers.

HPVs are DNA viruses that generate RNA transcripts for the expression of oncogenic proteins. The identification of high risk HPVs in TCGA prostate cancer samples by RNA-Seq indicates they are biologically active.

There is concern that the viral load is so low that HPVs may not be oncogenic in prostate cancer. It is relevant to note that similarly low HPV viral loads have been observed in some cervical cancers. A possible explanation is that the mechanisms by which HPVs act oncogenically in prostate cancer may be different from cervical and head and neck cancers.

Recent studies have shown that HPVs may have oncogenic mechanisms in addition to the influences of HPV E6 and E7 oncoproteins. These include APOBEC3B proteins which are a source of genome wide mutations and can lead to an increased risk of several cancers including prostate cancer [22]. APOBEC protein enzymes normally function as innate immune responses against viruses and other pathogens. The mechanisms of APOBEC–related mutational processes remained unknown, until the recent observations by Ohba et al. [22] and Vieira et al. [23] who independently demonstrated that infections with HPVs cause an overexpression of APOBEC3B protein. An additional mechanism for HPV associated oncogenesis has been investigated by Kundu et al. [24]. They demonstrated that components commonly present in HPVs and other pathogens can influence Toll-like receptor pathways and contribute to the malignant transformation of benign prostate epithelia.

The observation in this current study that HPV E7 oncoprotein is expressed at higher levels in benign prostate tissues as compared to subsequent prostate cancer in the same patients is consistent with HPVs acting early in prostate oncogenesis. This pattern of high prevalence of HPV E7 oncoprotein expression in benign prostate specimens and low expression in later prostate cancer specimens does not appear to be due to an inability of prostate cancer cells to express proteins as the expression of PSA was strong in many of the HPV E7 negative prostate cancer specimens. The implication is that HPVs may influence oncogenesis early in the development of prostate cancer.

This same phenomenon has been observed with HPV associated breast cancer [24]. This may be the HPV “hit and run” phenomenon previously described by others, whereby HPV infected cells transiently acquire a complete or incomplete viral genome in the early development of cancer but the virus becomes undetectable in the later stages of cancer [25]. This contrasts with the causal role of HPV in cervical cancer in which HPVs are required for both the initiation and maintenance of oncogenesis. This apparent involvement of HPVs at an early stage of prostate oncogenesis may explain the extremely low HPV viral load in fully developed prostate cancers. As demonstrated in this study HPVs are clearly not needed for maintenance of the prostate cancer.

## Conclusions

This study confirms that high risk HPVs are present in benign prostate tissues prior to the development of HPV positive prostate cancer in the same patients. These HPVs are biologically active and not harmless “passenger” viruses in prostate tissues. In addition, the much higher prevalence of HPV E7 oncoprotein expression in benign prostate tissues as compared to subsequent prostate cancers in the same patients suggests that HPV oncogenic activity is an early phenomenon in prostate oncogenesis.

## Additional files

Additional file 1: Table S1. HPV identification in the TCGA prostate cancer series. (XLS 12457 kb)

Additional file 2: Table S2. HPV sequences, HPV E7 protein, cytokeratin protein, PSA protein in benign prostate and subsequent prostate cancer in the same patients. (XLS 42 kb)
